# Preliminary Pharmacogenetic Study to Explore Putative Dopaminergic Mechanisms of Antidepressant Action

**DOI:** 10.3390/jpm11080731

**Published:** 2021-07-27

**Authors:** Taichi Ochi, Natalya M. Vyalova, Innokentiy S. Losenkov, Diana Z. Paderina, Ivan V. Pozhidaev, Anton J. M. Loonen, German G. Simutkin, Nikolay A. Bokhan, Bob Wilffert, Svetlana A. Ivanova

**Affiliations:** 1 PharmacoTherapy, Epidemiology and Economics, Groningen Research Institute of Pharmacy, University of Groningen, Antonius Deusinglaan 1, 9713AV Groningen, The Netherlands; t.ochi@rug.nl (T.O.); b.wilffert@rug.nl (B.W.); 2 Tomsk National Research Medical Center of the Russian Academy of Sciences, Mental Health Research Institute, Aleutskaya Str., 4, 634014 Tomsk, Russia; natarakitina@yandex.ru (N.M.V.); innokenty86@mail.ru (I.S.L.); osmanovadiana@mail.ru (D.Z.P.); craig1408@yandex.ru (I.V.P.); ggsimutkin@gmail.com (G.G.S.); bna909@gmail.com (N.A.B.); ivanovaniipz@gmail.com (S.A.I.); 3 Department of Cytology and Genetics, National Research Tomsk State University, Lenin Ave., 36, 634050 Tomsk, Russia; 4 Department of Psychotherapy and Psychological Counseling, National Research Tomsk State University, Lenin Ave., 36, 634050 Tomsk, Russia; 5 Department of Psychiatry, Addictology and Psychotherapy, Siberian State Medical University, Moskovsky Trakt, 2, 634050 Tomsk, Russia; 6 Department of Clinical Pharmacy and Pharmacology, University Medical Center Groningen, Hanzeplein 1, 9713GZ Groningen, The Netherlands; 7 Division for Control and Diagnostics, School of Non-Destructive Testing and Security, National Research Tomsk Polytechnic University, Lenin Ave., 30, 634050 Tomsk, Russia

**Keywords:** dopaminergic pathway, depression, antidepressants, mood disorders, dopamine, pharmacogenetics

## Abstract

Background: There is sufficient evidence that interference of dopaminergic neurotransmission contributes to the therapeutic effects of antidepressants in unipolar and bipolar depression. Methods: Hamilton depression rating scale (HAMD 17) scores of 163 at least moderately ill patients with major depressive disorders were used to establish treatment response. HAMD 17 score status was measured before initiation, after two weeks, and after four weeks of treatment with various antidepressants. The possible association between response and genotype in a total of 14 variants of dopamine neurotransmission-related proteins was investigated. Results: *DRD4* rs11246226 CA heterozygous patients were found with a greater improvement of HAMD 17 score when compared to homozygous C patients during 0–2 weeks and 0–4 weeks. Patients with *MAOB* rs1799836 heterozygous GA and homozygous A also demonstrated improved scores during 2–4 weeks and 0–4 weeks. Conclusions: The results are preliminary due to the limited population size and the small number of variants. Further research into the involvement of habenular dopamine D4 receptors in the antidepressant response is desirable.

## 1. Introduction

Depression, a leading cause of disability worldwide, can be treated with antidepressant drugs with a variety of pharmacological activities [1]. Antidepressants increase the extracellular concentration of monoamines within the brain by inhibiting their reuptake transporters (e.g., tricyclic antidepressants) or by catabolizing enzyme monoamine oxidase (e.g., MAO inhibitors). Targeting this etiological pathway derives from the monoamine hypothesis of depression, where depression stems from the deficiency in transmissions in monoamine systems [2]. Two of the monoamines, serotonin and norepinephrine, have been the main focus for antidepressant drug development, whereas dopamine has been somewhat neglected in this regard [3]. By focusing on dopaminergic neurotransmission, it may provide further insight into the personalised management of antidepressant medication [4,5].

Midbrain dopaminergic neurons stimulate fibres running through to the ventral striatum (accumbens nucleus), where the activity of ‘circuits regulating pleasure and happiness’ is modified [6]. Previously, these circuits been hypothesised to have a major role in causing core symptoms of unipolar and bipolar depressive disorders [7,8,9]. Pharmacological treatment with direct and indirect acting dopamine agonists has demonstrated therapeutic effects, especially in refractory or bipolar depression [10]. Direct agonists (bromocriptine, pramipexole, aripiprazole, cariprazine) are known to bind to dopamine (DA) receptors and activate them [11,12]. Oppositely, indirect agonists (bupropion, modafinil, tranylcypromine, entacapone) activate DA receptors by increasing the DA concentration [13,14]. These considerations lead to the expectation that genetic variation of the activity of DA structures may affect the effects of antidepressant drugs. Since the different monoaminergic systems interact to a great extent, this may well also apply to non-dopaminergic active antidepressants [5,15].

Five distinct but closely related DA receptor types can be distinguished, all five belonging to G protein-coupled receptors (GPCRs) and are divided into two major groups: the D1 type (DRD1, DRD5) and D2 type (DRD2, DRD3, DRD4) classes of dopamine receptors [16]. Particularly DRD1, DRD2, and DRD3 may be of interest for antidepressant effects as these are highly expressed in limbic cortical areas and the ventral striatum. Monoamine transporters, including dopamine transporters (DAT), the serotonin (SERT), and norepinephrine transporters (NET), are part of the solute carrier 6 (SLC6) family, which also includes the glycine transporter, GABA transporter, and neutral amino acid transporters [17]. *SLC6A3,* located on chromosome 5, encodes the DAT protein and plays a dominant DA regulatory role in the (ventral) striatum [18]. However, in the frontal cortex, the activity is lower than that of NET, which can transport dopamine as effectively as norepinephrine. This implies that altered catechol-O-methyltransferase (COMT) activity has much less influence in the (ventral) striatum than in the frontal cortex.

An antidepressant target, the MAO enzyme exists in two isoforms, both of which occur in the brain and for which two different X-bound genes encode (*MAOA* and *MAOB*) [19]. MAO-A has the most affinity for serotonin and norepinephrine, while MAO-B catabolizes phenylethylamine and benzylamine especially readily. Dopamine is broken down by both isoforms in humans, but it has relatively low affinity implying the influence of functional polymorphisms of *MAOA* cannot be considered specific for dopamine.

Pharmacogenetic investigations of the dopaminergic pathway have found associations in patients with Parkinson’s disease and schizophrenia, among other psychiatric disorders [20,21]. However, for depression, contradictory findings have greyed the landscape to determine whether polymorphisms impact antidepressant efficacy [22,23,24].

In this paper, we investigated single nucleotide polymorphisms (SNPs) in genes within the mesolimbic dopaminergic pathway against antidepressant response within the Tomsk cohort. We previously studied a possible relationship between genetic variants involved in serotonergic neurotransmission and the response to antidepressants in depressed patients who were free of treatment for at least six months. Our investigation explores the possible relationship between dopaminergic neurotransmission-related genes and antidepressive response in the cohort patients.

## 2. Materials and Methods

### 2.1. Patient Characteristics

The study design and patient characteristics have been described in an earlier study conduct with the cohort [25]. Newly admitted patients with a depressive episode according to the criteria of ICD-10 (F32 or F33) who had not been on antidepressant medication for at least 6 months were recruited [26]. More than half of them had never been treated with antidepressant drugs during their entire life. An increased number of patients were genotyped compared to previous studies, as an additional expansion of the scope of the study. As patients were seen by psychiatrists, the information pertaining to patient characteristics was limited to the depression scoring and basic information.

The patients’ depression was of at least moderate severity as measured by the Hamilton’s depression rating scale (HAMD 17) [27,28]. After this initial examination, an antidepressant treatment of at least four weeks was initiated and patients were re-examined with HAMD 17 after two and four weeks. For details on the inclusion and exclusion criteria of the patients, please refer to the earlier study in Ochi et al. [25]. The medication taken by patients consisted of mainly selective serotonin reuptake inhibitors (SSRIs) (Supplementary Table S1). The study complied with the Declaration of Helsinki (1975, revised in Fortaleza, Brazil, 2013) and was submitted and authorized by the Ethics Committee of the Mental Health Research Institute, Tomsk National Research Medical Center of the Russian Academy of Sciences (protocol 49 from 23.04.12). All patients were recruited from psychiatric departments of this institute and provided written informed consent.

### 2.2. Genotyping

Blood samples were drawn from antecubital venepuncture in evacuated EDTA tubes and aliquots were stored at −20 °C. DNA was isolated from leucocytes using the standard phenol-chloroform micro method. Polymorphisms in genes pertaining to the dopaminergic pathway were genotyped in the Laboratory of Genetics of the University of Groningen with the MassARRAY^®^ System (Agena Bioscience™, San Diego, CA, USA) and in the Laboratory of Molecular Genetics and Biochemistry of the Mental Health Research Institute with ‘StepOnePlus’ (Applied Biosystems, Waltham, MA, USA).

### 2.3. Selection of Genotypes

Due to the number of patients in the study, a post hoc power calculation was conducted to determine the number of investigated SNPs. From the total of 53 available variants of genes involved in dopaminergic neurotransmission, 14 candidate SNPs were selected. The selected SNPS met the Hardy–Weinberg equilibrium, had a minor allele frequency (MAF) of >5%, and had been reported to be associated with neurological or psychiatric disorders (schizophrenia, alcoholism, autism, Parkinson’s disease) for further analysis. These polymorphisms were localised within *DRD1* (rs4532), *DRD2* (rs6275, rs1801028, rs6277, rs1076560), *DRD3* (rs3773678, rs324035, rs167771, rs6280), *DRD4* (rs3758653, rs11246226), *MAOB* (rs1799836), and *SLC6A3* (rs464049, rs40184) genes.

### 2.4. Statistical Analysis

To determine the effect of dopaminergic pathway genes to antidepressant response, the outcome was measured by the difference in HAMD 17 score between entry and two weeks of treatment (ΔHAMD 17, 0–2 weeks) after two and four weeks of treatment (ΔHAMD 17, 2–4 weeks) and entry and four weeks of treatment (ΔHAMD 17, 0–4 weeks). A higher ΔHAMD 17 score demonstrated better clinical outcome. Normal distribution was tested utilizing the P-P plot.

Patients characteristics, including age, sex, and HAMD 17 scores across the different study periods, were emphasized using descriptive statistics (Table 1). Univariate linear regression was conducted to determine the SNPs to include in the multiple linear regression. Multiple linear regression was conducted to identify the independent factors associated with ΔHAMD 17 between the three time periods, including age, sex, depression diagnosis, type of antidepressant taken, and selected SNPs (Table 2; Supplementary Tables S2 and S3). To factor in the different categories in antidepressants taken and SNP genotypes, dummy variables were generated to establish the effect in each variable. Statistical analysis was conducted with SPSS software (release 25.0). The significance level for descriptive statistics and univariate statistical tests were *p* < 0.05. Factoring in Bonferroni correction, the significance level for multiple linear regression was *p* < 0.0031. Power analysis was conducted post hoc utilizing G*Power.

## 3. Results

### 3.1. Patients

The depressed patient cohort consisted of 163 patients, 22 male and 141 female, aged 49.5 ± 10.9 (mean ± standard deviation). Summary statistics of the cohort are indicated in Table 1. Majority of patients took selective serotonin reuptake inhibitors (SSRIs). Over the course of four weeks, HAMD 17 scores were measured at initiation (24.1 ± 4.9), at two weeks (12.9 ± 5.0) and at four weeks (5.1 ± 3.9) for each patient. For the cohort, a difference in HAMD 17 scores was found to be 11.2 ± 4.4 between initiation and week two, 7.8 ± 5.3 between week two and week four, and 19.0 ± 5.3 throughout the entire study.

### 3.2. Response to Treatment

Of the three time period analyses, ΔHAMD 17 signified improvement in patients taking tricyclic antidepressants (mainly clomipramine) in comparison to SSRIs between 0–2 weeks and 0–4 weeks (B = 4.4, *p* = 0.001; B = 2.7, *p* = 0.012; B = 4.4, *p* = 0.001, respectively). *DRD4* rs11246226 CA heterozygous patients were found with a decrease in ΔHAMD score compared to homozygous C patients in 0–2 weeks and 0–4 weeks (B = −2.5, *p* = 0.07; B = −3.5, *p* = 0.002). Patients with *MAOB* rs1799836 heterozygous GA and homozygous A demonstrated improvement in ΔHAMD score at 2–4 weeks and 0–4 weeks (GA: B = 2.22, *p* = 0.022; B = 2.3, *p* = 0.01 | AA: B = 2.8, *p* = 0.005; B = 3.0, *p* = 0.01). At 0–2 weeks, patients who were homozygous C for *DRD4* rs3758653 had decreased ΔHAMD in comparison to homozygous T (B = −9.2, *p* = 0.004). However, at 2–4 weeks, the effect on ΔHAMD was reversed (B = 7.6, *p* = 0.016). A negative effect on ΔHAMD was found with *DRD2* rs6275 homozygous C patients in relation to homozygous T patients at 2–4 weeks (B = −2.2, *p* = 0.017). At 0–2 weeks, *SLC6A3* rs40184 homozygous A patients were associated with a decreased ΔHAMD (B = −3.7, *p* = 0.006). No other significant associations were determined.

## 4. Discussion

In this study, we investigated possible associations between dopaminergic neurotransmission genetic variants and antidepressant treatment response in treatment-free patients with a major depressive episode. We limited our investigations to select variants which may influence the functioning of these pathways, as associations with diseases were previously described [18,22,23]. Our cohort consisted mainly of women (86.5%) but no associations were determined that gender played a role in antidepressant response for our study. Furthermore, the major depression diagnosis (e.g., whether it is recurrent or not) did not impact the outcome of our study. This may stem from the patient population, as patients had not received antidepressant treatment in the previous six months, minimizing the impact of previous antidepressant treatment.

Our investigation found significant associations for variants of the *DRD2*, *DRD4*, *MAOB*, and *SLC6A3* gene across the different study period. The impact of *DRD4* rs11246226 and *MAOB* rs1799836 appears to be consistent across different study periods in our study. On the other hand, the associations between *DRD2* rs6275 and *SLC6A3* rs40184 were identified in one study period. Surprisingly, the influence of *DRD4* rs3758653 was opposite in consecutive study periods. The associations found in SNPs in genes encoding MAO-B and DAT are in line with the indications that dopaminergic transmission plays a role in the antidepressant response. These aforementioned enzymes are believed to influence the concentration of dopamine in the synaptic cleft.

From our results, SNPs in *DRD4* have been indicative of playing a role in antidepressant response. The dopamine D4 receptor is best known for the relative specificity of the atypical antipsychotic clozapine for this receptor and their relative lack in the ventral and dorsal striatum [29,30]. The D4 receptor is less expressed in the mammalian brain than the D2 and D3 receptors and is mainly found in the frontal cortex, the amygdala, the hippocampus, the globus pallidus, the hypothalamus, the thalamus, and the substantia nigra pars reticulata [16,31]. This is different with the lamprey, which is a representative of the oldest human vertebrate ancestors, and where the D4 receptor is found in the striatum, cortex-like structures, and also, in the habenula [32].

We have good reason to believe that the lamprey striatum can be seen as a precursor to the human nuclear amygdala [33]. Dopamine D4 receptor has been observed in the central nucleus of the amygdala as well as in other regions of the complex [34]. Significantly, in the basal nucleus, the expression of D4 receptors and not D1 and D2 receptors is higher in depressed patients in comparison to healthy subjects. It is also interesting that the dopamine D4 receptor appears to be involved in relapse to nicotine, cocaine, and amphetamine use in addiction [35]. This phenomenon is linked to the role of the lateral habenula in reward-oriented behaviour, which also likely plays a role in depression [6,36]. Norepinephrine released from adrenergic terminals and not dopamine has been suggested to stimulate these habenular dopamine D4 receptors [37]. This could be the route by which antidepressants with noradrenaline reuptake inhibiting activity could mediate certain antidepressant effects.

While our findings may shed light on the dopaminergic neurotransmission pathways, our research has some limitations. As the study lacked a control group, we were unable to conduct a case-control study for the associations and determine if the SNPs also played a role in depression. In addition, the diversity of antidepressants taken may have impacted the response seen in our patient cohort. As the patients were seen by psychiatrists and not by physicians, the patient records are focused on the depression score and improvement of the study period, rather than providing a comprehensive medical report. Building on our results, we would suggest to further investigate, include more clinically relevant information, and consider the contribution of dopamine D4 receptors to the response to antidepressants. As we hypothesize the habenuloid complex playing a role in the dopaminergic neurotransmission response, we would highlight a special focus in this interaction when expanding the scope of the study.

## 5. Conclusions

In this cohort study, we investigated the possible existence of associations between genotypes of variants of genes encoding dopaminergic neurotransmission proteins and the response to antidepressant treatment of patients with major depressive disorder who had for at least 6 months been off treatment. The results of this study are suggestive to explore further the impact *DRD4* rs11246226 has on antidepressant response. The associations found in *DRD2* rs6275, *MAOB* rs1799836, and *SLC6A3* rs40184 are also worth investigating further. Our findings add to our hypothesis that dopamine D4 receptors in the amygdala and in the habenula may play a role in the response to antidepressants.

## Figures and Tables

**Table 1:** Patient characteristics.

Characteristics	Depressed Cohort (*n* = 163)
Total Number of Patients (%)	
Male	22 (13.5%)
Female	141 (86.5%)
Age in Years (Mean ± S.D.)	49.5 ± 10.9
Male	49.3 ± 9.3
Female	49.5 ± 11.1
HAMD 17 score (Mean ± S.D.)	
At entry	24.1 ± 4.9
At 2 weeks	12.9 ± 5.0
At 4 weeks	5.1 ± 3.9
Type of Depressive Episode (%)	
Single	92 (56.4%)
Recurrent	71 (43.6%)

**Table 2:** Multiple linear regression of total depression cohort covariates (age, gender, diagnosis, type of antidepressant, selected dopaminergic genotypes) for the whole study period (0 to 4 weeks).

Baseline Predictors	B	95% CI	*p*-Value	Baseline Predictors	B	95% CI	*p*-Value
(Constant)	17.57	9.33, 25.82					
Age	−0.44	−2.72, 1.85	0.71				
Gender	−0.02	−0.09, 0.05	0.58				
Diagnosis	−0.80	−2.31, 0.71	0.295				
DRD1 SNPs				DRD4 SNPs			
rs4532 CT	1.38	−3.52, 0.6	0.20	rs3758653 TC	0.55	−3.16, 3.37	0.54
s4532 TT	1.46	−0.71, 3.47	0.18	rs3758653 CC	−9.22	−1.21, 2.32	0.004 *
				rs11246226 CA	−2.54	−15.53, −2.92	0.007 *
DRD2 SNPs				rs11246226 AC	−1.46	−4.39, −0.69	0.16
rs6275 TC	0.61	−1.04, 4.48	0.52				
rs6275 CC	1.19	−1.28, 2.5	0.20	MAOB Receptor SNPs			
rs1801028 CG	−1.67	−0.62, 3	0.27	rs1799836 GA	0.09	−0.7, 3.63	0.92
rs6277 CT	0.46	−4.63, 1.3	0.66	rs1799836 AC	0.25	−1.82, 2.01	0.80
rs6277 TC	−0.67	−1.59, 2.51	0.61				
rs1076560 CA	−1.32	−3.27, 1.93	0.21	SLC6A3 Receptor SNPs			
rs1076560 AA	0.76	−3.39, 0.75	0.78	rs464049 CT	0.12	−1.72, 2.21	0.90
				rs464049 TT	−0.08	−1.66, 1.9	0.94
DRD3 SNPs				rs40184 GA	−0.59	−2.16, 2	0.49
rs3773678 CT	0.46	−4.67, 6.19	0.77	rs40184 AA	−2.01	−2.3, 1.12	0.08
rs3773678 TT	0.35	−2.68, 3.61	0.92				
rs324035 CA	−2.09	−6.33, 7.03	0.41	Treatment (compared to SSRIs)			
rs324035 AA	−6.78	−7.09, 2.91	0.10	TCAs	2.70	0.62, 4.79	0.012 *
rs167771 GA	−3.86	−14.96, 1.4	0.36	SNRIs	0.41	−2.16, 2.98	0.75
rs167771 AA	−4.08	−12.1, 4.38	0.31	NaSSAs	1.08	−1.92, 4.09	0.48
rs6280 CT	0.51	−11.94, 3.77	0.62	Agomelatine	1.72	−1.04, 4.48	0.22
rs6280 CC	0.11	−1.5, 2.53	0.95				
						R-squared	
						0.26	

Data are presented as regression coefficients (B), 95% confidence intervals (CI) and total explained variance (r2); * *p* < 0.05; significance for *p* values after correction: ** *p* < 0.0031; *** *p* < 0.001; HAMD, Hamilton depression score rating difference; TCAs, tricyclic antidepressants; SNRIs, serotonin–norepinephrine reuptake inhibitors; NaSSAs, noradrenergic and specific serotonergic antidepressants.

## Data Availability

The datasets generated for this study will not be made publicly available, but they are available on reasonable request to Svetlana A. Ivanova (ivanovaniipz@gmail.com), following approval of the Board of Directors of the MHRI, in line with local guidelines and regulations.

## Supplementary Materials

The following are available online at https://www.mdpi.com/article/10.3390/jpm11080731/s1, Table S1: Antidepressant medication taken by patients; Table S2: Multiple linear regression of total depression cohort covariates (age, gender, diagnosis, type of antidepressant, selected dopaminergic genotypes) for the first two-week study period (0 to 2 weeks); Table S3: Multiple linear regression of total depression cohort covariates (age, gender, diagnosis, type of antidepressant, selected dopaminergic genotypes) for the second two-week study period (2 to 4 weeks).
