# Supplementary material for: Preliminary Pharmacogenetic Study to Explore Putative Dopaminergic Mechanisms of Antidepressant Action
Source: J Pers Med. 2021 Jul 27;11(8):731. doi: 10.3390/jpm11080731 (PMC8401614; doi:10.3390/jpm11080731)
Supplement: Supplementary file 1 [file jpm-11-00731-s001.zip › Ochi et al., 2021 - Supplementary Table 1.pdf]

Supplementary Table S1.—antidepressant medication taken by patients.

Class	Antidepressant	Patients
**SSRIs**	**Total**	**100**
	Sertraline	26
	Paroxetine	23
	Escitalopram	17
	Fluoxetine	14
	Fluvoxamine	12
	Citalopram	4
	Trazodone + Paroxetine	2
	Fluvoxamine + Agomelatine	1
	Trazodone	1
**TCAs**	**Total**	**25**
	Clomipramine	18
	Pipofezine	6
	Amitriptyline	1
**SNRIs**	**Total**	**15**
	Venlafaxine	11
	Duloxetine	4
**Agomelatine**	**Agomelatine**	**12**
**NaSSa**	**Total**	**11**
	Mirtazapine	7
	Mianserin	4

Antidepressants taken over the course of the four weeks by patients. SSRIs: selective serotonin receptor inhibitors, TCAs: tricyclic antidepressants; SNRIs: serotonin–norepinephrine reuptake inhibitors; NaSSAs: noradrenergic and specific serotonergic antidepressants.
