# Supplementary material for: Preliminary Pharmacogenetic Study to Explore Putative Dopaminergic Mechanisms of Antidepressant Action
Source: J Pers Med. 2021 Jul 27;11(8):731. doi: 10.3390/jpm11080731 (PMC8401614; doi:10.3390/jpm11080731)
Supplement: Supplementary file 1 [file jpm-11-00731-s001.zip › Ochi et al., 2021 - Supplementary Table 2.pdf]

Supplementary Table S2. Multiple linear regression of total depression cohort covariates (age, gender, diagnosis, type of antidepressant, selected dopaminergic genotypes) for the first two-week study period (0 to 2 weeks).

Baseline Predictors	B	95% CI	p-value	Baseline Predictors	B	95% CI	p-value
(Constant)	19.53	9.92 , 29.14					
Age	0.89	-1.78 , 3.56	0.51				
Gender	-0.01	-0.08 , 0.07	0.90				
Diagnosis	-1.45	-3.21 , 0.31	0.10				
<u>DRD1 SNPs</u>				<u>DRD4 SNPs</u>			
rs4532 CT	1.54	-0.89 , 3.98	0.21	rs3758653 TC	0.59	-1.47 , 2.64	0.57
rs4532 TT	0.63	-1.89 , 3.15	0.62	rs3758653 CC	-1.65	-9.01 , 5.7	0.66
				rs11246226 CA	-3.53	-5.69 , -1.38	0.002**
<u>DRD2 SNPs</u>				rs11246226 AA	-1.46	-3.78 , 1.03	0.26
rs6275 TC	-0.20	-2.4 , 2.01	0.86				
rs6275 CC	-0.98	-3.08 , 1.13	0.36	<u>MAOB Receptor SNPs</u>			
rs1801028 CG	-0.39	-3.85 , 3.07	0.82	rs1799836 GA	2.28	0.05 , 4.52	0.05
rs6277 CT	0.93	-1.46 , 3.31	0.45	rs1799836 AA	3.04	0.75 , 5.33	0.01*
rs6277 TT	-0.94	-3.97 , 2.09	0.54				
rs1076560 CA	-0.28	-2.7 , 2.13	0.82	<u>SLC6A3 Receptor SNPs</u>			
rs1076560 AA	2.10	-4.23 , 8.42	0.51	rs464049 CT	-1.27	-3.34 , 0.81	0.23
				rs464049 TT	-1.38	-3.8 , 1.04	0.26
<u>DRD3 SNPs</u>				rs40184 GA	-1.14	-3.13 , 0.85	0.26
rs3773678 CT	1.55	-2.12 , 5.22	0.40	rs40184 AA	-3.66	-6.26 , -1.05	0.01*
rs3773678 TT	5.50	-2.29 , 13.28	0.17				
rs324035 CA	-1.86	-7.69 , 3.98	0.53	<u>Treatment (compared to SSRIs)</u>			
rs324035 AA	-4.20	-13.73 , 5.34	0.39	TCAs	4.34	1.91 , 6.77	0.001**
rs167771 GA	0.83	-8.78 , 10.43	0.87	SNRIs	0.04	-2.95 , 3.04	0.98
rs167771 AA	0.61	-8.55 , 9.77	0.90	NaSSAs	-1.50	-5 , 2.01	0.40
rs6280 CT	0.65	-1.7 , 3	0.58	Agomelatine	-0.88	-4.1 , 2.34	0.59
rs6280 CC	-0.85	-4.66 , 2.95	0.66				
						R-squared	
						0.31	

Data are presented as regression coefficients (B), 95% confidence intervals (CI), and total explained variance (r2); * $p < 0.05$; significance for p values after correction: ** $p < 0.0031$; *** $p < 0.001$; HAMD: Hamilton depression score rating difference; TCAs: tricyclic antidepressants; SNRIs: serotonin–norepinephrine reuptake inhibitors; NaSSAs: noradrenergic and specific serotonergic antidepressants.
