# Supplementary material for: Preliminary Pharmacogenetic Study to Explore Putative Dopaminergic Mechanisms of Antidepressant Action
Source: J Pers Med. 2021 Jul 27;11(8):731. doi: 10.3390/jpm11080731 (PMC8401614; doi:10.3390/jpm11080731)
Supplement: Supplementary file 1 [file jpm-11-00731-s001.zip › Ochi et al., 2021 - Supplementary Table 3.pdf]

Supplementary Table S3. Multiple linear regression of total depression cohort covariates (age, gender, diagnosis, type of antidepressant, selected dopaminergic genotypes) for the second two-week study period (2 to 4 weeks).

Baseline Predictors	B	95% CI	p-value	Baseline Predictors	B	95% CI	p-value
(Constant)	1.96	-6.09 , 10.01					
Age	1.33	-0.91 , 3.56	0.24				
Gender	0.01	-0.05 , 0.08	0.68				
Diagnosis	-0.65	-2.12 , 0.82	0.384				
<u>DRD1 SNPs</u>				<u>DRD4 SNPs</u>			
rs4532 CT	0.17	-1.88 , 2.21	0.87	rs3758653 TC	0.03	-1.69 , 1.76	0.97
rs4532 TT	-0.83	-2.95 , 1.28	0.44	rs3758653 CC	7.57	1.41 , 13.73	0.016*
				rs11246226 CA	-0.99	-2.8 , 0.81	0.28
<u>DRD2 SNPs</u>				rs11246226 AA	0.09	-1.93 , 2.1	0.93
rs6275 TC	-0.81	-2.65 , 1.04	0.39				
rs6275 CC	-2.16	-3.93 , -0.4	0.02*	<u>MAOB Receptor SNPs</u>			
rs1801028 CG	1.28	-1.62 , 4.17	0.39	rs1799836 GA	2.19	0.32 , 4.06	0.022*
rs6277 CT	0.47	-1.53 , 2.47	0.64	rs1799836 AA	2.80	0.88 , 4.71	0.005**
rs6277 TT	-0.27	-2.81 , 2.27	0.84				
rs1076560 CA	1.04	-0.99 , 3.06	0.31	<u>SLC6A3 Receptor SNPs</u>			
rs1076560 AA	1.34	-3.96 , 6.63	0.62	rs464049 CT	-1.39	-3.12 , 0.35	0.12
				rs464049 TT	-1.30	-3.33 , 0.72	0.21
<u>DRD3 SNPs</u>				rs40184 GA	-0.55	-2.22 , 1.12	0.51
rs3773678 CT	1.09	-1.98 , 4.16	0.48	rs40184 AA	-1.65	-3.83 , 0.54	0.14
rs3773678 TT	5.15	-1.37 , 11.67	0.12				
rs324035 CA	0.24	-4.65 , 5.12	0.92	<u>Treatment (compared to SSRIs)</u>			
rs324035 AA	2.59	-5.4 , 10.57	0.52	TCAs	1.64	-0.4 , 3.68	0.11
rs167771 GA	4.69	-3.36 , 12.73	0.25	SNRIs	-0.37	-2.88 , 2.14	0.77
rs167771 AA	4.69	-2.98 , 12.36	0.23	NaSSAs	-2.58	-5.51 , 0.35	0.08
rs6280 CT	0.14	-1.83 , 2.11	0.89	Agomelatine	-2.60	-5.3 , 0.09	0.06
rs6280 CC	-0.96	-4.15 , 2.23	0.55				
						R-squared	
						0.31	

Data are presented as regression coefficients (B), 95% confidence intervals (CI) and total explained variance (r2); * $p < 0.05$; significance for p values after correction: ** $p < 0.0031$; *** $p < 0.001$; HAMD: Hamilton depression score rating difference; TCAs: tricyclic antidepressants; SNRIs: serotonin–norepinephrine reuptake inhibitors; NaSSAs: noradrenergic and specific serotonergic antidepressants.
